# Antibiotics and lectin C for diarrhea control intervention in piglets and influences

**DOI:** 10.1186/s13568-024-01775-4

**Published:** 2024-11-14

**Authors:** Hoang Dinh Trung, Ha Viet Hoang, Nguyen Thach Thong, Kenthalangsy Chitana, Dinh Thi Thu Hoai, Nguyen Quang Linh

**Affiliations:** 1 Faculty of Biology, University of Sciences, 77 Nguyen Hue St., Hue City, 530000 Vietnam; 2 Department of Animal Husbandry, Faculty of Animal Science and Veterinary Medicine of Biology, University of Agriculture and Forestry, Hue University, Hue City, 490000 Vietnam

**Keywords:** Antibiotics, Lectin C recovery, Death rate, Infected rate and reinfected

## Abstract

The study was conducted on 60 L of suckling piglets out of 775 piglets, of which 227 piglets had diarrhea litres of piglets, and out of 775 piglets, 227 piglets had diarrhoea syndrome. There were 3 interventions in separate trials as follows: 1 and 2 involved antibiotic use (Enrofloxacin and Gentamicin); Trial 3 used the supplement Lectin C (LvCLT3 and LvCLT4) for a treatment period of 3–7 days. Intervention results on 227 piglets with diarrhea showed the highest cure rate when supplemented with Lectin C, with no mortality rate, longer treatment time, and no reinfection rate. While the two antibiotic trials still had lower cure rates, mortality and reinfection rates were higher. After 05 days of recovery, piglets were tested for MCV, MCH, and MCHC, showing an apparent decrease in the group supplemented with Lectin C, while the two groups using antibiotics still had high rates, with a confidence level of *P* < 0.05; 0.01 and 0.001. Antibiotics treat piglets in their early stages, so there is a risk of immunodeficiency and low infection response. It is necessary to supplement substances derived from dietary supplements. Supplementing Lectin C increases resistance, enhances immune response, improves the effectiveness of treating diarrhoea syndrome in piglets, and ensures safe meat quality in the future. Lectin C supplementation will improve piglet health and breed quality efficiency. This heralds a promising future for the pig industry with improved meat quality and reduced environmental impact.

## Introduction

Diarrhea in piglets is a pervasive issue in pig farms, particularly in industrial pig farms. Piglet diarrhea syndrome, occurring from birth to weaning, is a disease that inflicts substantial economic losses on pig breeding facilities. The disease’s epidemiological characteristics are complicated, with the rate of piglet diarrhea in farms/farming households ranging from 30 to 60% (Pliszczak-Król et al. 2016), intensive pig farms also have a higher rate of diarrhoea. If left untreated, it can lead to stunting and slow growth, hampering the breed’s potential to gain weight and causing significant economic harm. However, the potential financial benefits of preventing diarrhea in piglets are immense. Pig breeding facilities can significantly improve their financial performance by enhancing breeding efficiency and ensuring a steady supply of high-quality breeds. Piglet diarrhea is a syndrome that stems from a multitude of causes, a complex interplay of various factors, both internal and external: Genetic factors, microorganisms or parasites, and other factors such as temperature and humidity. Among these, the key to proactive prevention lies in the resistance of piglets through passive antibodies (Shousong et al. 2020) and active antibodies through vaccines. This knowledge forms the basis of our project: ‘Diarrhea in suckling piglets and testing the effectiveness of the treatment regimen, (Shousong et al. 2020). The study aimed to 1) assess the prevalence of diarrhea syndrome in suckling piglets at the farm and 2) conduct testing of treatment regimens and evaluate their effectiveness, especially applying the new Lectin C orally. Our ultimate goal is to recommend these interventions for widespread application. This mission holds great significance for the future of piglet health and breeding efficiency and offers a promising outlook for the industry.

## Materials and methods

### Materials and experimental animals

There were 227 piglets with diarrhea out of 775 piglets in 60 litters by the same pig farm and breed, a crossbreed between PIDUx LY (Pietrain x Duroc) and F1 sows (Landrace x Yorkshire). According to Dak Ha Pig Farm, all piglets were raised under the same breeding conditions and supplemented with creep feed from 07 days old until weaning at 21 days. All of the piglets at the farm had injected Fe^++^ in the form of Dextral Fe, 20%, 1 ml for each at 3 days old.

### Used drugs for treating diarrhea syndrome

- *Type APA Enro I*: Product of APA Japan Nano Technology Co., Ltd. (Enrofloxacin, 10 g and solvent, excipients q.s. 100 ml), drug specifically for treating pasteurellosis, atrophic rhinitis, typhoid, swollen head caused by E.coli, secondary infections after viral infections caused by Gram-positive and Gram-negative bacteria sensitive to Enrofloxacin such as *E.coli*, *Mycoplasma*, *Salmonella*, S*taphylococcus*, *Pasteurella*, *Streptococcus*. Injection dose: 1 ml/15kg body weight for piglets in intervention 1, trial 1.
- *Gentamycin 10%*: Product of Veterinary Medicine Production and Trading Company Limited (Gentamycin (sulfate) 100 mg and solvent for injection sufficient for 1 ml intramuscular injection continuously for 3–5 days, dose of 1 ml/20 kg for piglets in intervention 2, trial 2.
- *Lectin C*: produced according to the State-level Project granted by the Ministry of Science and Technology, Code: DTĐL.CN-56/22 contains LvCTL3 and LvCTL4 to supplement piglets, chickens, and shrimp. In intervention 3, trial 3, Lectin C is used at a dose of 0.05 g for 1 kg of creep feed for piglets.

### Experimental design

- Experimental design and time: The experiment was conducted at Dak Ha pig farm, Dak Nong province, from February 5, 2023, to April 27, 2023, according to Table 1, which shows the context of the experiment.
- Variables and criteria: ObservationsObservations of infected and diarrheal piglets; treatment days; recovery and dead rate; blood variables (MCV, MCH, MCHC) on each unit used; reinfected rate and mortality rate.
- Method and care: Piglets with diarrhea are marked separately for monitoring and treatment. Each herd will apply the treatment regimen and monitor the pigs in the early morning and afternoon before and after using the drug. Monitor the treatment results daily until the pigs no longer show signs of diarrhea syndrome. Spray green pen to mark piglets that have recovered. Continue to monitor the treated animals to know the number of animals that relapse and the number of animals that die. The farm recorded indicators of the rate of piglet diarrhea with the naked eye and clinical symptoms on the piglet’s body. Observe the faeces on the barn floor where the piglets often defecate (brown, yellow, milky white and liquid faeces, etc.). Observe the pigs in the pen if there is a rotten smell or signs of diarrhea. If the child has a stool stuck in the anus or wet bottom, check and treat. Observe the piglets’ physical condition and expressions for clinical diagnosis. Decreased physical condition, thinness, ruffled fur, moody appearance, often lying in the womb, etc., check and treat. The mother pig’s condition also affects the piglets’ health and condition. Mother pigs stop eating, have ruffled hair, become thin, have mastitis, metritis, etc., causing milk quality to decline and piglets not being provided with enough nutrients, making it easy for microorganisms to invade and cause diarrhoea. Determine the Incidence of Diarrhea by Stage of Development:
- Record information and data: sow ears, number of piglets with diarrhea syndrome, treatment regimen, date of illness, date of recovery, number of dead pigs, date of death, and number of relapsed pigs. Summary of monitoring results and treatment effectiveness of the regimen. Blood variables: Blood biochemical indices such as red blood cells, white blood cells, heamoglobin and hematocrit, mean corpuscular volume (MCV), mean corpuscular hemoglobin (MCH): mean corpuscular heamoglobin concentration (MCHC): Average hemoglobin concentration in a red blood cell and changes in red blood cell size is taken and determined according to the piglet sample in the herd. Blood is taken from the jugular vein of the piglet early in the morning before feeding and exercise. Each time, 1 ml of blood is taken into a test tube containing the anticoagulant (NaHCO_3_, 1 ml), shaken well, put in a styrofoam box, and transported to the Department of Hematology, Institute of Biotechnology, Hue University for analysis. The number of red blood cells, white blood cells, hemoglobin, and hematocrit were determined by an automatic cell counter SYSMEX KX 21 (Japan).

**Table 1:** Experimental design and setting

Trails	1	2	3
Number of litters	20	20	20
Number of young piglets	258	258	259
Infected piglets	75	72	80
Medicines	APA Enro I	Gentamycin 10%	Lectin C
Major antibiotics	Enrofloxacin	Gentamycin	Protein
Description and Doses	0,3 − 0,5 ml/muscular infection/1 time/day	00,3 − 0,5 ml/muscular infection/1 time/day	0,05 g/kg feed
Treatment days	3– 5	3– 5	5–7

### Data and analysis

All variables were recorded by day of experiments and analysis by Excel and Anova test with *P* < 0.05 for 3 stages of growing suckling piglets: 0–7, 8–14, and 15–21 days.

### Test of two treatment protocols

- Antibiotic control interventions:

1. Intervention 1 - trial 1: APA Enro I, monitoring piglets with diarrhea in 20 pens in farrowing pen (1) Treatment with protocol 1.
2. Intervention 2 - trial 2: Gentamycin 10%, monitoring piglets with diarrhea in 20 pens in farrowing pen (2) Treatment with protocol 2.
3. Intervention 3 - trial 3: Supplementation (LvCTL3 and LvCTL4), monitoring piglets with diarrhea in 20 pens in farrowing pen, supplementing 0.05 g Lectin C/kg feed and feed for piglets fed with Lectin C.

## Results

### Prevalence of piglet diarrhea syndrome at Dak ha pig farm

**Fig. 1:**
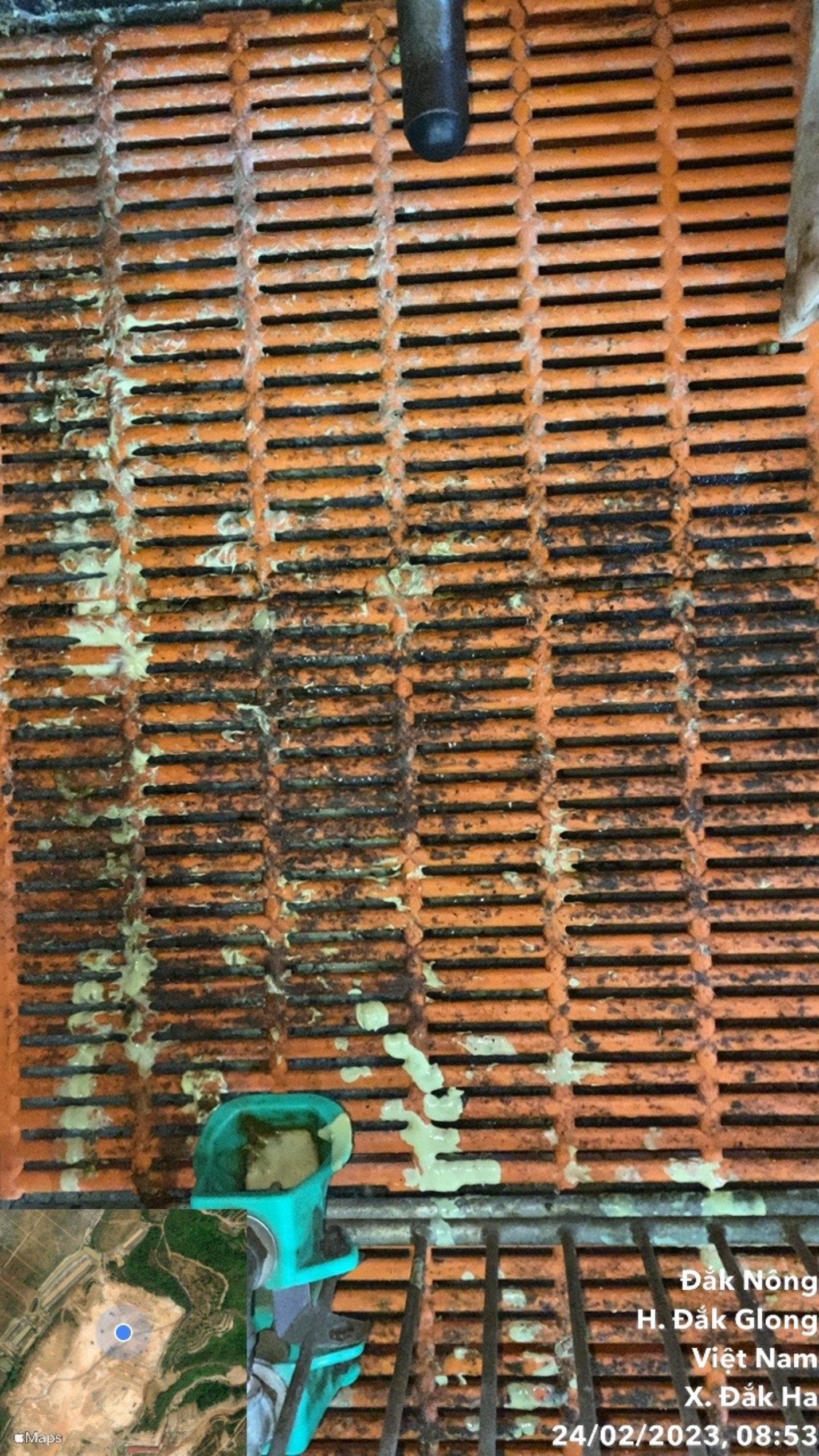
Piglets have diarrhea on floor

**Fig. 2:**
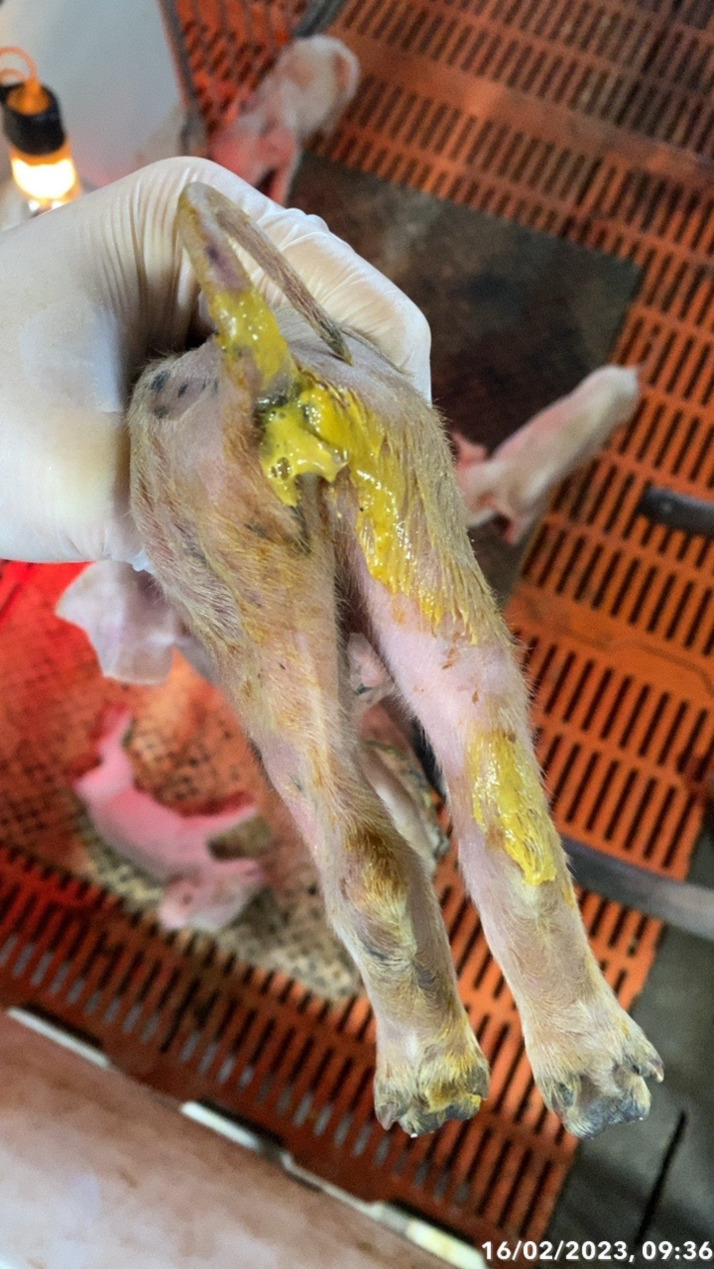
Acure signs of diarrhea on piglets

To better understand the damage caused by diarrhea syndrome in suckling piglets and have a scientific basis for providing disease prevention and treatment measures at Dak Ha pig farm, we surveyed 60 litters of piglets, with 775 piglets, 227 piglets were sick, with signs of observing faeces on the barn floor where piglets often defecate (brown, yellow, milky white and liquid faeces). Observation of piglets on the floor, the signal symptom in Figs. 1 and 2, there is a foul odor or signs of diarrhea, and the piglet has faeces stuck in the anus or a wet bottom. We obtained results on the incidence of diarrhea syndrome in piglets, as shown in Table 1, and signs of diarrhea in piglets. Table 2 shows that the rate of piglets with diarrhea syndrome is 29.29%.

**Table 2:** Infected percentages in farms

Number of piglets (*n*)	Diarrhea-infected piglets(*n*)	Infected rates (%)
775	227	29.29

### Percentage of piglets with diarrhea syndrome over months

During the internship at the pig farm, we directly monitored the diarrhea situation in piglets following their mothers in the three months of February, March, and April 2023 at the Dak Ha pig farm in the highlands. Table 3 shows that the incidence of diarrhea syndrome between the three months is different: April, with 31.68%, is higher than February by 26.92%, and March, at 29.25%. February and March have similar diarrhea incidence rates, with a difference of 0.51%. The temperature drops at night, leading to an extensive temperature range between day and night. When the humidity increases, the increased temperature will inhibit the body’s heat dissipation process, causing thermal imbalance and reducing the body’s resistance. At the same time, the constantly changing weather makes it difficult for piglets to adapt and combined with reduced resistance, it causes a high rate of disease in March. Although diarrhea in April (31.68%), followed by March (29.25%), the humidity is not high because many sows give birth during this time, and piglets lose milk, leading to diarrhea.

**Table 3:** Results of diarrhea rates from February toApril 2023

Variables	Number of piglets (*n*)	Number of infected piglets(*n*)	Infected rates (%)
February	260	70	26.92
March	253	74	29.25
April	262	83	31.68
Average	775	227	29.29

### Percentage of piglets with diarrhea syndrome according to age stages

The rate of diarrhea in piglets depends on external factors such as microorganisms, care regime, weather and age of the pig. In evaluating the diarrhea situation in piglets according to age, we monitored 60 blood-sampled piglets of 227 from 775 initial piglets (20 piglets per batch monitored by intervention 1 (trail 1) and blood sampling, 20 piglets monitored by intervention 2 (trail 2) and 20 individuals monitored by intervention 3 (trail 3) respectively. The piglets were from the same litter, had similar average birth weights and had the same feeding and care regimen. Table 4 and Fig. show the difference in the incidence of diarrhea in piglets between age groups. The incidence of diarrhea in piglets from 8 to 14 days old was the highest (27.02%), followed by 15 to weaning (8.36%), 0 to 7 days old (4.40%).

**Table 4:** Infected percentage by ages

Ages (weeks)	Number of piglets (*n*)	Infected piglets (*n*)	Infected rates %
0 to 7	775	34	4.40
8 to 14	775	209	27.02
15 to weaning	754	63	8.36

### Results using 3 disease control interventions

Diarrhea in piglets occurs for many reasons, but regardless of the cause, the final and most common cause is bacteria that play a major or minor role, mainly *E.coli* and other factors. The most important of which is *E.coli*. Diarrhea often leads to dehydration and cardiovascular collapse, causing piglets to lose weight and die. Therefore, when treating diarrhea in piglets, it is necessary to combine treatment of the cause of the disease with symptomatic treatment, improve the health of piglets, protect the intestinal mucosa, and prevent intestinal dysbiosis that leads to stunting later. To contribute to finding effective prevention and treatment measures, we used two different treatment regimens to choose the better treatment regimen. The results of testing two regimens on piglets from birth to 28 days of age are shown in Table 5. After using the drug, the rate of re-infected piglets was 14.06% and 19.35%, respectively, compared to the control intervention with Enrofloxacin and Gentamicin. The number of pigs dying when using both regimens was relatively 11.11% and 17.33% for the two control interventions with antibiotics, Enrofloxacin and Gentamicin, the mortality rate was 11.11%. In Control Intervention 2, the mortality rate was higher at 17.33%. Enrofloxacin using APA Enro I was the cure rate, and 89.46% of piglets recovered. In regimen 2 (using Gentamycin), there were no piglet deaths, and Lectin C, with 100% of piglets recovery after 5 days.

**Table 5:** Results of two treatment medicines (interventions)

Variables/Interventions	1	2	3
Number of treatment piglets (n)	72	75	80
Number of recovered piglets (n)	64	62	80
Recovered rate (%)	89.46	82.67	100
Re-Infected piglets (n)	9	12	0
Re-infected rates (%)	14.06%	19.35%	0
Dead piglets (n)	8	13	0
Dead rates (%)	11.11%	17.33%	0

The most common blood tests, MCV, MCH, and MCHC, were examined as important health variables in the suckling period. However, with a bit of insight, changes in blood, and different tests of different control interventions for piglet healthcare decisions for using medicine or supplementation, such as Lectin C. Mean corpuscular volume (MCV), no significant differences were found between different control interventions (Table 6).

**Table 6:** Piglet blood variables after treatment 5 days, the red blood cell (RBC) indices are the

Age in days (*n* = Number of piglets)	MCV (fL)	MCH (pg)	MCHC (%)
Trail 1 (*n* = 30/72)	69.24^**^ ± 5.71	22.85 ± 2.07	32.99 ± 1.12
Trail 2 (*n* = 30/75)	72.16^**^ ± 5.58	19.72^**^ ± 1.74	30.66 ± 0.62
Trail 3 (30/80)	57.89^***^ ± 4.69	17.98^***^ ± 1.28	31.12^*^ ± 1.43

Refers: **, with *P* < 0.01 and *** with *P* < 0.001 significant differences berween trials

## Discussions

Tables 1 and 2 explain that, firstly, the climatic and epidemiological characteristics of different months of the year are different due to monthly climate changes. Secondly, the ability to receive antibodies through breast milk is different due to the different ages of piglets during the suckling period (Pliszczak-Król et al. 2016; Faustini et al. 2003). However, diarrhea is a pathological syndrome related to many factors; some are the leading causes, and some are secondary causes, so vaccination and disease prevention only bring specific effects. Therefore, diarrhea in piglets following the mother still occurs frequently on farms, where although the amount of iron is fully supplemented for the whole herd, the diarrhea rate is still high. This result is consistent with the study (Dao Trong Dat et al. 2000) published that the disease rate in concentrated pig farms is 20–50% (Luong et al. 2022) study on the diarrhoea rate is 50%,. Our survey results are still much lower than (Shousong et al. 2020), up to 75–82%. The difference in the results of the rate of piglets with diarrhea between seasons and months and the age of the pig is due to the different research facilities limiting the diarrhea rate. The incidence of diarrhea in piglets from birth to 7 days of age is lower than that of piglets from 8 to 14 days of age (Yoshioka 2020) reported that because this is the stage when piglets are entirely dependent on their mothers, the impact of harmful microorganisms has been limited by antibodies through breast milk, this result was also published by (Catherine 2019). The main effects on piglets are climate, weather, surrounding conditions and food, especially breast milk, such as Gama Globulin, as mentioned by (Shousong et al. 2020). In general, this result is somewhat different from the studies of some other authors, such as (Dao Trong Dat et al. 2000); Healthy piglets at different weeks of age have different rates of diarrhea, and at week 2, especially from 10 to 14 days, the rate of diarrhea is highest (27.02%), followed by week 3, from 15 to 21 days old which is also high (8.16%) and week 1, from birth to 7 days old (4.40%). Research by (Doan Kim Dung, 2004) showed that the rate of diarrhea in pigs in the period of 8 to 14 days was 34.11% when monitoring 170 piglets, much higher than our results. According to the results of (Luong et al. 2022), our piglets have the highest rate of diarrhea in the age group of 8–14 days old (37.12%) and the lowest rate of diarrhea in piglets from birth to 7 days old is only 7.40% and the lowest is in the age group from 15 days to weaning (14.73%). The difference in disease incidence between age groups is related to physiological changes occurring inside the piglets’ bodies and the impact of the external environment, especially the vaccination program for pregnancy sows.

The difference in disease incidence between age groups is related to physiological changes occurring within the piglet’s body and the impact of the external environment, especially the *E.coli*vaccination program for pregnant sows in the late stage after 90 days of gestation and the very high antibody content in colostrum. Piglets are suckled with colostrum as soon as possible after birth, so the mother’s body transmits passive immune factors to fight against adverse environmental factors. Specific antibodies in colostrum such as Gama globulin, Vitamins A, E, C and D, MgSO4, and intestinal mucosa prevent bacteria from adhering to receptors on the surface of the small intestinal epithelium and neutralize the activity of bacterial enterotoxins and cytotoxins. Therefore, colostrum is piglets’ main immunity source in the first week after birth. In addition, iron accumulates in the body from the fetal period, iron from breast milk, and supplementary iron (through supplementary injections), sufficient to supply the piglet’s body. Therefore, the resistance of piglets is better and more stable than that of the 8–14 day-old stage (Faiborther 1992; Kalai et al. 2012; Perri AM. 2015). The disease incidence from 15 days old to weaning is much lower than that of the period from 8 to 14 days old. Piglets at this stage gradually adapt to environmental conditions, strengthen and improve the body’s resistance. On the contrary, by the 3rd week, piglets have begun to eat to compensate for nutritional deficiencies; the nervous system is also developing, sensing, and regulating thermogenesis to warm the body or radiate heat to release body heat. Therefore, we can limit the cause of diarrhea caused by E. coli in 3-week-old piglets and piglets preparing to wean. From the 3rd week of growth, the growth rate of piglets begins to slow down because the amount of breast milk secreted decreases. Supplemental food plays an important role. Combining it with a Lectin C dose, which is lower than the therapeutic dose, can be highly effective. The amount of minerals (mainly iron) in breast milk and piglets’ reserves is insufficient (Doan 2004) and is enough to meet the needs. Blood indices in 60 piglets were monitored, with results consistent with the data (Cooper et al. 2014), and found that the mean MCV ranged from 66 to 69.4 fL according to (Angel et al. 2014), finding significant differences in MCH and MCHC treatment in different drugs may have different blood indices as reported by (Angel M.A. et al. 2014). However, piglets given Lectin C showed different levels of drug (Gentamicin and Enrofloxacin) treatment was lower in piglets as reported by Budak et al. (2016). Lower MCV values ​​(64.8–64.9 fL) were noted. The mean cell hemoglobin concentration (MCH), as well as the mean cell hemoglobin concentration (MCHC) in our trial (Table 6), were slightly more significant than the mean values ​​obtained by another author (Cooper et al. 2014). They stated the ranges of MCH and MCHC as 19.4–21.8 pg and 30.2–31.0%, respectively, since the authors recorded similar MCV values ​​(71.55 ± 5.88fL). The difference in disease incidence between age groups is related to physiological changes occurring inside the piglets’ bodies and the impact of the external environment, especially the vaccination program for pregnancy sows. On the other hand, the antibody content in colostrum is very high. Piglets are fed colostrum immediately after birth, so the mother’s body transmits passive immune factors to fight against adverse environmental factors. Specific antibodies in colostrum or intestinal mucosa prevent bacteria from adhering to receptors on the surface of the small intestinal epithelium and neutralize the activity of enterotoxins and bacterial cytotoxins. Therefore, colostrum is piglets’ main immunity source in the first week after birth. In addition, iron accumulates in the body from the fetal period, iron from breast milk, and supplementary iron (through supplementary injections), sufficient to supply the piglet’s body. Therefore, the resistance of piglets is better and more stable at 8–14 days of age (Faiborther 1992; Kalai et al. 2012; Perri 2015). The incidence of disease is much lower in the period from 15 days of age to weaning than in the period from 8 to 14 days of age. Piglets at this stage gradually adapt to environmental conditions, strengthen and improve their body’s resistance. On the contrary, by the third week, piglets have begun to eat to compensate for nutritional deficiencies, and the nervous system is also developing. Therefore, we can limit the cause of diarrhea caused by E. coli in 3-week-old piglets. In the third week of growth, the growth rate of piglets begins to slow down because the amount of milk the mother produces decreases. The amount of minerals (mainly iron) in the mother’s milk and the piglets’ reserves are insufficient (Doan 2004) and are sufficient to meet the needs. The results are consistent with the data (Cooper et al. 2014), which found that the average MCV was 66–69, also conducted the range of MCH and MCHC as 19.4–21.8 pg and 30.2–31.0%, respectively, since similar MCV values ​​(71.55 ± 5.88fL) were noted by (Willis et al. 2021), while the trail results showed at supplemented Lectin C for piglets, all of 3 indicators decreased with values: 15.89fL, 17.98pg and 31.12% respectively, benefited for piglets and growth will be much better postweaning stage.

## Data Availability

Ethics approval and consent to participate: All animals and samples were applied to international, national, regional, and institutional guidelines for animal care and rules in Vietnam. Ethical Certificate of HUAF for The uses of all animals and samples in the study applied international, national, regional and institutional guidelines for animal care and rules in Vietnam: 10.6084/m9.figshare.22736792.

## Abbreviations

PCR: Polymerase chain reaction
TCBS: Thiosulphate citrate bile salt sucrose
tlh: Thermolabile hemolysin toxin
trh: Tdh-related hemolysin
tdh: Thermostable direct hemolysin
toxR: Toxin operon
